# Limb salvage in diabetic patients with no-option critical limb ischemia: outcomes of a specialized center experience

**DOI:** 10.1080/2000625X.2019.1696012

**Published:** 2019-11-28

**Authors:** Luca Dalla Paola, Paolo Cimaglia, Anna Carone, Giuseppe Scavone, Giulio Boscarino, Davide Bernucci, Paolo Sbarzaglia, Stefano Censi, Roberto Ferrari, Gianluca Campo

**Affiliations:** a Maria Cecilia Hospital, GVM Care & Research, E.S.: Health Science Foundation, Cotignola, Italy; b Cardiovascular Center, Azienda Ospedaliero-Universitaria di Ferrara, Cona, Italy

**Keywords:** Diabetic foot ulcer, limb salvage, critical limb ischemia, limb amputation, diabetic foot surgery

## Abstract

**Objective**: To describe the characteristics, the management and the outcome of a consecutive series of patients with diabetic foot lesions (DF) and no-option critical limb ischemia (CLI) treated with a multidimensional, interdisciplinary approach in a dedicated center.

**Research Design and Methods**: The prospective database of the Diabetic Foot Unit of the Maria Cecilia Hospital (Cotignola, Italy) collects medical history, risk factors, chemistry values, angiographic data, characteristic of foot lesions, medical and surgical therapies of all patients admitted with a diagnosis of DF and CLI. All patients were followed-up for at least 1 year and/or total recovery. The primary endpoint was 1-year amputation-free survival (AFS), secondary endpoints were limb salvage and survival.

**Results**: Between October 2014 and October 2017, 1024 patients with DF and CLI were admitted to the center. Eighty-four of them (8.2%) fulfilled the criteria for no-option CLI. At 1 year, AFS, limb salvage, and survival rates were 34%, 34%, and 83%, respectively. Lesions located proximal to the Lisfranc joint were associated with major amputation (HR 2.1 [1.2–3.6]). One-year survival of patients treated with minor procedures was significantly higher compared to patients treated with major amputation (96% vs 76%, log-rank p = 0.019). Major amputation was independently associated with mortality (HR 7.83 [1.02–59.89]).

**Conclusions**: The application of dedicated and standardized strategies permitted limb salvage in one-third of patients with no-option CLI. Patients with stable lesions limited to the forefoot and without ischaemic pain had a greater probability to successfully receive conservative treatments. Limb salvage was associated with subsequent higher one-year survival.

Diabetic foot (DF) lesions are a major health problem. It concerns 9 to 26 million persons, it is costly and responsible for 70% of non-traumatic amputations in the western world [1–4]. In addition to that, amputation is also associated with a high mortality rate and social and psychological problems [5–8]. DF is often associated with critical limb ischemia (CLI), the end-stage symptomatic evolution of peripheral artery disease [9,10]. Revascularization through percutaneous transluminal angioplasty (PTA) or through bypass graft surgery is of paramount importance for wound healing and limb salvage [11]. However, revascularization is not always possible. The terms ‘no-option’ and ‘non-reconstructable’ CLI have been used for decades to identify those patients unsuitable for revascularization [12]. Today, thanks to the technological improvements especially for endovascular treatment, the percentage of patients not suitable for reperfusion has been decreasing and, as a consequence, the amputation-free survival of CLI patients is improving [13]. Nevertheless, these cases still exist and represent a very high-risk category. Their management is difficult, and the major amputation often seems to be considered the only available option.

This article aims to challenge this concept by describing the characteristics, the management and the outcome of a consecutive series of patients with DF and no-option CLI treated with a multidimensional, interdisciplinary approach in a dedicated center.

## Research design and methods

Clinical and procedural data from all patients admitted to the Diabetic Foot Unit of the Maria Cecilia Hospital (Cotignola, Italy) are recorded in a dedicated clinical database and accurately verified for completeness and accuracy against the patients’ clinical charts. Patients are prospectively followed up for at least 12 months. The analysis was based on current clinical practice; therefore, the regulatory authorities required an ordinary written informed consent to procedures and data collection, which was obtained from all patients. The protocol of the study was in accordance with the Declaration of Helsinki.

### Definitions and study population

For the definition of diabetes, DF, CLI and no-option CLI, current standards and guidelines were followed. In short, diabetes was diagnosed according to Standards of Medical Care in Diabetes criteria [14]; DF was diagnosed in presence of non-healing ulcers or gangrene below the ankle; CLI was defined as presence of chronic ischemic rest pain and/or ulcers or gangrene (consistent with Fontaine Stages III-IV and Rutherford Classes 4 to 6) attributable to proven arterial occlusive disease [15]. Artery disease was assessed through transcutaneous oxygen tension (<30 mm Hg) and imaging (Doppler examination and/or angiography). A patient was judged no-option CLI when considered ineligible for surgical or endovascular revascularization by a ‘foot team’ including a diabetologist/foot surgeon (LDP), a cardiologist (AC), an interventional cardiologist (PS) and a vascular surgeon (GB). The population of the present analysis consists of 84 patients with DF and no-option CLI. Patients with acute limb-threatening ischemia, trauma, non-atherosclerotic disease (e.g. arteritis), embolic disease, or known hypercoagulable states were excluded.

### Multidimensional and interdisciplinary approach

The diagnostic and therapeutic approach of DF patients in the Diabetic Foot Unit of the Maria Cecilia Hospital follows an ad hoc designed internal protocol consistent with the international guidelines and recommendations. Firstly, patients with infected ulcers are treated with antibiotics guided by microbial culture, and in case of acute infection (abscess, phlegmon, wet gangrene), urgent surgical incision/drainage is performed. Secondly, the ischemic aetiology of DF is ascertained with duplex scanning ultrasound and transcutaneous oxygen tension measurement; when positive, angiography and PTA are performed. In case of unsuccessful PTA or of anatomy not suitable for PTA, the indication to bypass graft surgery is considered with the surgical team. For those with no indication to surgery (no-option CLI), our ad hoc clinical protocol starts, with the endpoint of maintaining the plantar standing. Patients with no pain at rest and stable lesions are considered for conservative management or minor amputation. Conservative management consists of ulcerectomy and, in case of osteomyelitis (detected by plain x-rays and/or magnetic resonance), sequestrectomy. The closure is carried out through primary intention with gentle procedures, carefully avoid any tension on surgical margins, using nylon monofilament sutures 3–0 or 4–0. The subcutaneous suture is not used. In case this approach cannot be adopted, negative pressure wound therapy (V.A.C. ®, Acelity) is applied, followed by the application of dermal substitute (Integra® Dermal Regeneration Template, Integra LifeSciences Corporation). Subsequently, a split-thickness skin graft is used to cover the residual wound. In the case of minor amputation, the level is established according to the location and extension of necrosis. Finally, at discharge, antidiabetic and cardiovascular preventive therapies are optimized and off-loading of the index limb is strongly recommended. During follow-up, all patients with a healed surgical site start a secondary prevention program consisting of the prescription of custom-molded, individually designed shoes and insoles, and periodic medical, orthotic and podiatry visits. Only the patients with pain at rest not responsive to pharmacological therapy and/or progression of infection/necrosis are treated with major amputation.

### Follow-up and endpoints

Patients returned for follow-up visits every 15 days until the clinical stabilization of DF and surgical site. Afterwards, they were visited every 2 months. During the visits, patients were examined and assessed for adverse events and compliance to medical therapy. Additional exams and tests were performed at the physician’s discretion. The primary outcome analysed is the one-year amputation-free survival (AFS), defined as freedom from death or major amputation, whichever occurred first. Major amputation was defined as any amputation above the ankle. Limb salvage and survival rates are also reported to ascertain the main driver of the combined endpoint. Source documents of adverse events were collected and reviewed by an independent blinded reviewer (PC) for the final adjudication.

### Statistical analysis

Continuous data were tested for normal distribution with the Kolmogorov–Smirnov test and are presented as mean ± SD or median and interquartile range as appropriate. The normally distributed variables were compared using a t-test and a one-way ANOVA; otherwise, the Mann–Whitney U and Kruskal–Wallis tests were used. Categorical variables are summarized in terms of numbers and percentages and were compared using the two-sided Fisher’s exact test. All variables were tested using univariate Cox regression as predictors of major amputation or death. Variables showing a p-value <0.1 were included in the multivariable model. Independent predictors among baseline characteristics were selected with a backward stepwise modelling approach. The variables remaining significant at a threshold p-value ≤0.05 were retained as final predictors. Finally, the cumulative occurrence of AFS in patients stratified according to the presence or not of baseline foot lesions located distal to the Lisfranc joint and of mortality in patients stratified according to the presence or not of major amputation were assessed using Kaplan–Meier curves. Differences between groups were judged with the log-rank test. A p-value was considered significant if <0.05. All analyses were performed with SPSS 24.

## Results

Between October 2014 and October 2017, 1,024 patients with DF and CLI were admitted to our Unit. Overall, 1,981 angiography of lower limbs were performed in this group of patients. In all the patients an endovascular or surgical procedure was carried out but in 84 patients (8.2%) there was a failure in distal revascularization with a persistent CLI after procedures. After a discussion in the multidisciplinary **‘**foot team**’**, those patients were judged affected by DF with no-option CLI and represent the study population of the current investigation.

### Baseline characteristics

Table 1 shows the main characteristics of the study population. The median age was 77 [70–84] years, and 58 (69%) patients were male. Ischemic heart disease (55%), atrial fibrillation (36%), heart failure (19%) and end-stage renal disease (19%) were the more commonly observed comorbidities (Table 1). As expected, the majority of patients had a history of revascularization of the lower limbs and minor or major amputations. The main details of DF at presentation are reported in Table 2. Thirty-five percent of lesions were located proximal to the Lisfranc joint, and 56% showed gangrene. The median transcutaneous oxygen tension was 7 [3–14] mmHg. The angiography showed occlusion of all the arteries below the knee in 36 (43%) patients, while only five (6%) had no total occlusive disease below the knee. About pedal arteries, 70 (83%) patients had both dorsal and plantar artery occluded. In the remaining 14, one of the two main arteries was angiographically patent, without corresponding benefit assessed by transcutaneous oximetry (probably due to reduced perfusion).

**Table 1.** Characteristics of the study population and univariate analysis.

	Population (n = 84)	1-year major amputation or death HR (95% CI)
Age, (years)	77 [70–84]	0.997 (0.971–1.024)
Male sex, no. (%)	58 (69)	1.540 (0.838–2.831)
BMI, (kg/m^2^)	23.9 [22.0–26.1]	0.996 (0.931–1.065)
Hypertension, no. (%)	60 (71.4)	0.735 (0.426–1.268)
Dyslipidemia, no. (%)	66 (78.6)	0.831 (0.486–1.421)
Type 1 diabetes, no. (%)	3 (3.6)	0.663 (0.092–4.799)
**Comorbidities**		
Diabetic retinopathy, no. (%)	16 (19.0)	0.942 (0.445–1.994)
Carotid artery disease, no. (%)	15 (17.9)	0.693 (0.339–1.417)
Stroke, no. (%)	7 (8.3)	1.700 (0.724–3.991)
Heart failure, no. (%)	16 (19.0)	0.724 (0.354–1.480)
Ischemic heart disease, no. (%)	46 (54.8)	1.175 (0.687–2.010)
Atrial fibrillation, no. (%)	30 (35.7)	0.706 (0.401–1.242)
Valvular prosthesis, no. (%)	12 (14.3)	1.077 (0.527–2.203)
ESRD on hemodialysis, no. (%)	16 (19.0)	1.295 (0.680–2.465)
COPD, no. (%)	11 (13.1)	1.486 (0.698–3.166)
Rheumatoid arthritis, no. (%)	12 (14.3)	0.906 (0.410–2.002)
**Laboratory data**		
Hemoglobin, (g/dl)	10.0 [9.3–11.1]	0.780 (0.611–0.995)
White blood cells, (10^3^/μL)	10.4 ± 3.4	1.081 (0.998–1.171)
Platelets, (10^3^/μL)	304 [230–384]	1.001 (1.000–1.003)
Creatinine clearance, (ml/min)	54.5 [27.9–87.3]	1.002 (0.995–1.009)
Cholesterol, (mg/dl)	136.9 ± 37.0	0.991 (0.983–0.999)
HDL, (mg/dl)	32.0 [27.0–44.0]	0.983 (0.960–1.006)
Triglycerides, (mg/dl)	122.5 [97.3–156.3]	0.998 (0.992–1.004)
LDL, (mg/dl)	69.2 [51.8–101.2]	0.991 (0.982–1.001)
Hemoglobin A_1c_, (mmol/mol) (%)	51.0 [42.0–62.0] 6.8 [6.0–7.8]	0.979 (0.946–1.008)
CRP, (mg/dl)	5.0 [2.1–9.6]	1.043 (1.009–1.079)
**Previous PAD treatment**		
PTA, no (%)	78 (92.9)	0.464 (0.184–1.169)
Bypass surgery, no (%)	14 (16.7)	1.054 (0.531–2.092)
Amputation*, no (%)	53 (63.1)	1.342 (0.763–2.361)
**Medical therapy**		
Oral anticoagulants, no (%)	36 (42.9)	1.090 (0.640–1.859)
Aspirin, no (%)	76 (90.5)	1.336 (0.482–3.698)
Clopidogrel, no (%)	60 (71.4)	0.908 (0.512–1.610)
ACEi/ARBs, no (%)	27 (32.1)	0.763 (0.430–1.353)
Beta-blockers, no (%)	49 (58.3)	0.976 (0.571–1.669)
Statins, no (%)	61 (72.6)	1.068 (0.590–1.933)
High-potency statins†, no (%)	15 (17.9)	1.109 (0.559–2.203)
Ezetimibe, no (%)	4 (4.8)	1.585 (0.570–4.405)
Insulin therapy, no. (%)	67 (79.8)	0.888 (0.425–1.856)
Oral hypoglycemic drugs, no. (%)	15 (17.9)	0.988 (0.439–2.227)

ESRD, end-stage renal disease; COPD, chronic obstructive pulmonary disease; CRP, C-reactive protein; PAD, peripheral artery disease; PTA, percutaneous transluminal angioplasty; ACEi, angiotensin-converting enzyme inhibitors; ARBs, angiotensin receptor blockers.

*Any kind of amputation in either leg.

†Atorvastatin 40/80 mg or rosuvastatin 10/20/40 mg.

**Table 2.** Characteristics of the index lesion and univariate analysis.

	Population (n = 84)	1-year major amputation or death HR (95% CI)
**Location**		
Digits, no (%)	17 (20.2)	
Forefoot, no (%)	38 (45.2)	
Midfoot, no (%)	4 (4.8)	
Hindfoot, no (%)	7 (8.3)	
Ankle, no (%)	3 (3.6)	
Diffuse foot involvement, no (%)	11 (13.1)	
Leg/residual limb, no (%)	4 (4.8)	
Proximal to Lisfranc joint, no (%)	29 (34.5)	2.080 (1.221–3.545)
**Characteristics**		
Plantar lesion, no (%)	6 (7.2)	1.120 (0.404–3.102)
Gangrene, no (%)	47 (56.0)	2.483 (0.338–18.240)
TcPO2, (mmHg)	7 [3–14]	1.001 (0.969–1.035)
**Angiographic data**		
N° of patent tibial arteries:		
3	5 (6.0)	
2	15 (17.9)	
1	28 (33.3)	
0	36 (42.9)	
N° of patent pedal arteries:		
2	0 (0)	
1	14 (16.7)	
0	70 (83.3)	

TcPO2, transcutaneous oxygen tension.

### Amputation-free survival

**Figure 1.**
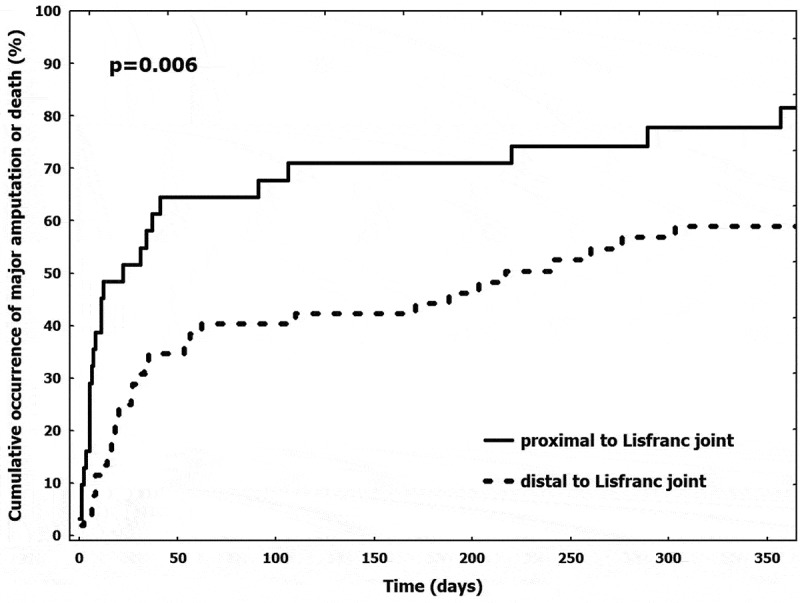
Occurrence of major amputation or death in patients with lesions located proximal or distal to the Lisfranc joint.

One-year follow-up was available in all patients, whilst a longer follow-up (median 916 [584–1218] days) was available in 55 (65%) patients. AFS rate was 34% at 1 year and 27% in the overall available follow-up. Hence, 23 (27%) patients resulted alive and with the index limb salvaged at the end of the study. Their amputation-free survival was 708 [383–1073] days. AFS rate had significantly increased in patients with baseline foot lesions located distal to Lisfranc joint compared to patients with lesions proximal to it, both at 1-year (42% vs 19%, log-rank p = 0.006) (Figure 1) and during the overall follow-up (40% vs 7%, log-rank p < 0.001). At univariate analysis, variables associated with major amputation or death at one-year were foot lesion location (proximal vs distal to Lisfranc joint, HR 2.080 [1.221–3.545]), total cholesterol (HR 0.991 [0.983–0.999]), C-reactive protein (HR 1.043 [1.009–1.079]) and hemoglobin (HR 0.780 [0.611–0.995]). After multivariable analysis, lesion location and total cholesterol were independent predictors of AFS.

### Limb salvage

One year after the diagnosis of no-option CLI, 55 (66%) patients received a major amputation of the index limb, corresponding to a limb salvage rate of 34%. The level of the amputation was above-the-knee in 22 (40%) and below-the-knee in 33 (60%) patients. The median time between no-option CLI diagnosis and major amputation was 20 [6–62] days. In 62% of cases, the major amputation was performed within 1 month from diagnosis. Variables associated with one-year major amputation were lesion location (proximal versus distal to Lisfranc joint, HR 2.101 [1.233–3.580]), total cholesterol (HR 0.991 [0.983–0.999]), C-reactive protein (HR 1.040 [1.005–1.076]) and hemoglobin (HR 0.780 [0.611–0.995]). After multivariable analysis, lesion location and total cholesterol remained independent predictors of major amputation. Overall, all patients with successful limb salvage received a minor amputation. Minor amputation was performed at midfoot, transmetatarsal and digits level in 5 (21%), 14 (58%) and 5 (21%) patients, respectively.

### Survival

**Figure 2.**
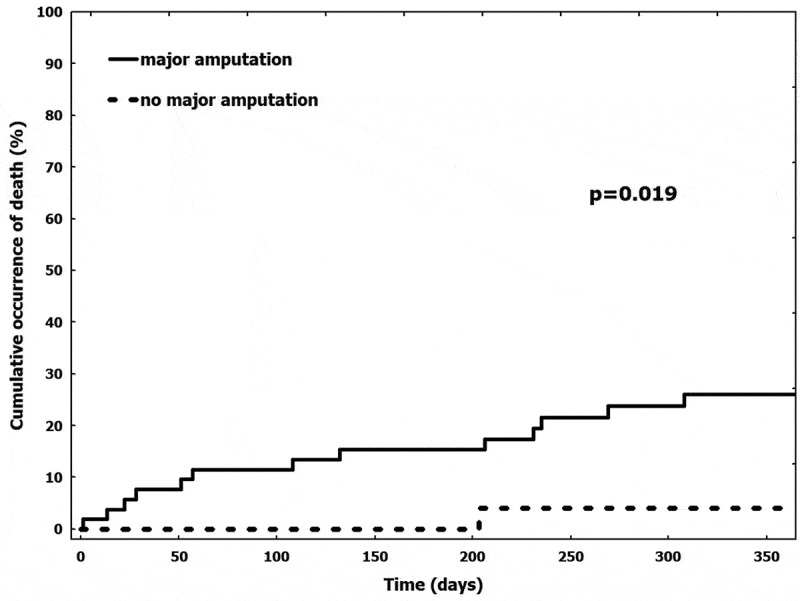
Occurrence of death in patients treated with major amputation and in those who had a successful limb salvage.

At 1 year, 14 (17%) patients died, corresponding to a survival rate of 83%. The median time between no-option CLI diagnosis and death was 145 [72–257] days. Thirteen (93%) patients underwent a major amputation of the index limb before death. One-year mortality of patients treated with major amputation was significantly higher compared to that in patients who had a successful limb salvage (24% vs 4%, log-rank p = 0.019) (Figure 2). The median time between major amputation and death was 74 [58–230] days. No survival differences were noted between patients treated with below- versus above-the-knee amputation (log-rank p = 0.932). The only variable associated with 1-year mortality was major amputation (HR 7.832 [1.024–59.889]).

## Conclusions

The present analysis, despite the limitations inherent to its design, has demonstrated that major amputation is not mandatory for patients with DF and no-option CLI.

In as much as 34% of our consecutive population, we avoided amputation above or below the knee in the first year, and this positive result was maintained in 27% of the patients subjected to the long-term follow-up. The rate of limb salvage reported in clinical trials is higher, but the most critical cases were often excluded, as well as patients on dialysis [16–19]. Furthermore, even the definition of no-option CLI adopted in these trials are variable and subjective [13]. There are few real-world data on no-option CLI patients for comparison. In a recent retrospective study, patients that received the diagnosis of no-option CLI had a limb salvage rate of 0% at 1 year [20]. Faglia et al. reported 27 cases of no-option CLI with amputation free-survival lower than 10% at 1 year [8]. Thus, the results obtained with the present study are encouraging and make this approach clinically relevant.

The clinical parameters that allowed us to consider the limb salvage attempt were: the absence of ischemic pain at rest; the stability of the chronic lesions without progression of infection. Conversely, in all cases with ischemic pain and progression of the soft tissue destruction, major amputation had to be carried out. At the regression analysis, the strongest predictive variable associated with limb salvage was the location of the index lesions distal to the Lisfranc joint. As one might expect, it was easier to treat lesions that are more distal and to maintain the plantar standing. Indeed, transmetatarsal amputation was the most commonly performed minor amputation in patients with successful limb salvage. This founding was also confirmed in the study by Baer-Bositis et al., which showed a negative association between heel ulcers and amputation-free survival [20].

Our approach could be of interest to the field also because the no-option CLI patients who had a successful attempt of limb salvage showed a significantly higher survival rate than those treated with major amputation. Moreover, at the multivariate analysis, major amputation has been confirmed as an independent predictor of mortality. The evidence that mortality increases after major amputation is not new and is well described in the medical literature. However, this study shows for the first time that avoiding major amputation leads to a survival benefit also in the high-risk group of no-option CLI patients.

Besides major amputation, no other clinical variable or risk factor associated with mortality has been identified. It appears that the amputation itself enhanced the risk of death, rather than being a marker of the underlying severity of the disease. Nevertheless, no statistically significant conclusion can be made given the size of our population sample. It is relevant to consider that decision-making for amputation procedures is subjective and complex. In our protocol, rest pain and control of infection/necrosis were the main determinants to decide whether to perform a limb salvage attempt. However, previous ambulatory status, as well as predicted function of the limb, comorbidities, frailty and patient’s life expectancy were all considered in the final clinical decision. Such variables were not always available for our analysis and should be considered in future studies.

In conclusion, the main finding of the present study is that a limb salvage procedure is feasible and effective even in patients with DF and no-option CLI, and especially in those with lesions located in the forefoot. A successful limb salvage attempt was associated with improved survival. Considering that the mortality rate after major amputation in the diabetic population is substantial, our study prompt to pursue the limb salvage attempt even in the high-risk group of no-option CLI.

## Article highlights

**Type of Research**: Single-center retrospective analysis of prospectively collected registry data.

**Key Findings**: Amputation-free survival of 84 consecutive no-option CLI patients was 34% at 1 year. Patients with lesions located proximal to the Lisfranc joint had a higher incidence of major amputation and subsequent higher mortality rate.

**Take-home Message**: Limb salvage can be achieved in one-third of no-option CLI patients, and this may lead to improved survival.

## Table of Contents Summary

In this retrospective analysis of 84 no-option CLI patients, a successful limb salvage attempt was associated with improved survival. The authors suggest to pursue the limb salvage in no-option CLI patients, especially in those with lesions located in the forefoot.

## References

[1] Armstrong DG, Boulton AJM, Bus SA. Diabetic foot ulcers and their recurrence. N Engl J Med. 2017;376:1–7. 28614678 10.1056/NEJMra1615439

[2] Almaraz MC, González-Romero S, Bravo M, et al Incidence of lower limb amputations in individuals with and without diabetes mellitus in Andalusia (Spain) from 1998 to 2006. Diabetes Res Clin Pract. 2012;95:399–405. 22133651 10.1016/j.diabres.2011.10.035

[3] Trautner C, Haastert B, Spraul M, et al Unchanged incidence of lower-limb amputations in a German City, 1990-1998. Diabetes Care. 2001;24:855–859. [cited 2018 1124]. Available from: http://www.ncbi.nlm.nih.gov/pubmed/11347743 11347743 10.2337/diacare.24.5.855

[4] Rice JB, Desai U, Cummings AKG, et al Burden of diabetic foot ulcers for medicare and private insurers. Diabetes Care. 2014;37:651–658. 24186882 10.2337/dc13-2176

[5] Thorud JC, Plemmons B, Buckley CJ, et al Mortality after nontraumatic major amputation among patients with diabetes and peripheral vascular disease: a systematic review. J Foot Ankle Surg. 2016;55:591–599. 26898398 10.1053/j.jfas.2016.01.012

[6] Shah SK, Bena JF, Allemang MT, et al Lower extremity amputations: factors associated with mortality or contralateral amputation. Vasc Endovascular Surg. 2013;47:608–613. 24005190 10.1177/1538574413503715

[7] Hoffstad O, Mitra N, Walsh J, et al Diabetes, Lower-Extremity amputation, and death. Diabetes Care. 2015;38:1852–1857. 26203063 10.2337/dc15-0536

[8] Faglia E, Clerici G, Caminiti M, et al Mortality after major amputation in diabetic patients with critical limb ischemia who did and did not undergo previous peripheral revascularization. Data of a cohort study of 564 consecutive diabetic patients. J Diabetes Complications. 2010;24:265–269. 19328013 10.1016/j.jdiacomp.2009.02.004

[9] Monteiro-Soares M, Boyko EJ, Ribeiro J, et al Predictive factors for diabetic foot ulceration: a systematic review. Diabetes Metab Res Rev. 2012;28:574–600. 22730196 10.1002/dmrr.2319

[10] Faglia E Characteristics of peripheral arterial disease and its relevance to the diabetic population. Int J Low Extrem Wounds. 2011;10:152–166. 21856972 10.1177/1534734611417352

[11] Faglia E, Dalla Paola L, Clerici G, et al Peripheral angioplasty as the first-choice revascularization procedure in diabetic patients with critical limb ischemia: prospective study of 993 consecutive patients hospitalized and followed between 1999 and 2003. Eur J Vasc Endovasc Surg. 2005;29:620–627. 15878541 10.1016/j.ejvs.2005.02.035

[12] Rümenapf G, Morbach S What can i do with a patient with diabetes and critically impaired limb perfusion who cannot be revascularized? Int J Low Extrem Wounds. 2014;13:378–389. 25326447 10.1177/1534734614554283

[13] Benoit E, O’Donnell TF, Kitsios GD, et al Improved amputation-free survival in unreconstructable critical limb ischemia and its implications for clinical trial design and quality measurement. J Vasc Surg. 2012;55:781–789. 22209608 10.1016/j.jvs.2011.10.089

[14] American Diabetes Association AD Diagnosis and classification of diabetes mellitus. Diabetes Care. 2014;37(Suppl 1): S81–90. 24357215 10.2337/dc14-S081

[15] Norgren L, Hiatt WR, Dormandy JA, et al Inter-society consensus for the management of peripheral arterial disease (TASC II). J Vasc Surg. 2007;45:S5–S67. 17223489 10.1016/j.jvs.2006.12.037

[16] Klomp HM, Spincemaille GH, Steyerberg EW, et al Spinal-cord stimulation in critical limb ischaemia: a randomised trial. ESES Study Group. Lancet. 1999;353:1040–1044. [cited 2018 1128]. Available from: http://www.ncbi.nlm.nih.gov/pubmed/10199350 10199350 10.1016/s0140-6736(98)05069-7

[17] Amann W, Berg P, Gersbach P, et al European peripheral vascular disease outcome study SCS-EPOS. Spinal cord stimulation in the treatment of non-reconstructable stable critical leg ischaemia: results of the European peripheral vascular disease outcome study (SCS-EPOS). Eur J Vasc Endovasc Surg. 2003;26:280–286. [cited 2018 1128]. Available from: http://www.ncbi.nlm.nih.gov/pubmed/14509891 14509891 10.1053/ejvs.2002.1876

[18] Nikol S, Baumgartner I, Van Belle E, et al Therapeutic angiogenesis with intramuscular NV1FGF improves amputation-free survival in patients with critical limb ischemia. Mol Ther. 2008;16:972–978. 10.1038/mt.2008.33 28178491

[19] Marston WA, Davies SW, Armstrong B, et al Natural history of limbs with arterial insufficiency and chronic ulceration treated without revascularization. J Vasc Surg. 2006;44:108–114.e1. 16828434 10.1016/j.jvs.2006.03.026

[20] Baer-Bositis HE, Hicks TD, Haidar GM, et al Outcomes of reintervention for recurrent symptomatic disease after tibial endovascular intervention. J Vasc Surg. 2018;68:811–821.e1. 29525414 10.1016/j.jvs.2017.11.096

